# ASIC subunit ratio and differential surface trafficking in the brain

**DOI:** 10.1186/s13041-016-0185-7

**Published:** 2016-01-08

**Authors:** Junjun Wu, Yuanyuan Xu, Yu-Qing Jiang, Jiangping Xu, Youjia Hu, Xiang-ming Zha

**Affiliations:** Department of Physiology and Cell Biology, University of South Alabama College of Medicine, 5851 USA Dr N, MSB3074, Mobile, AL 36688 USA; China State Institute of Pharmaceutical Industry, 285 Gebaini Road, Shanghai, 201203 China; School of Pharmaceutical Sciences, Southern Medical University, Guangzhou, China; Department of Urology, The Third Hospital of Hebei Medical University, Shijiazhuang, HeBei China

**Keywords:** ASIC, Subunit ratio, Stoichiometry, Surface expression

## Abstract

**Background:**

Acid-sensing ion channels (ASICs) are key mediators of acidosis-induced responses in neurons. However, little is known about the relative abundance of different ASIC subunits in the brain. Such data are fundamental for interpreting the relative contribution of ASIC1a homomers and 1a/2 heteromers to acid signaling, and essential for designing therapeutic interventions to target these channels. We used a simple biochemical approach and semi-quantitatively determined the molar ratio of ASIC1a and 2 subunits in mouse brain. Further, we investigated differential surface trafficking of ASIC1a, ASIC2a, and ASIC2b.

**Results and conclusions:**

ASIC1a subunits outnumber the sum of ASIC2a and ASIC2b. There is a region-specific variation in ASIC2a and 2b expression, with cerebellum and striatum expressing predominantly 2b and 2a, respectively. Further, we performed surface biotinylation and found that surface ASIC1a and ASIC2a ratio correlates with their total expression. In contrast, ASIC2b exhibits little surface presence in the brain. This result is consistent with increased co-localization of ASIC2b with an ER marker in 3T3 cells. Our data are the first semi-quantitative determination of relative subunit ratio of various ASICs in the brain. The differential surface trafficking of ASICs suggests that the main functional ASICs in the brain are ASIC1a homomers and 1a/2a heteromers. This finding provides important insights into the relative contribution of various ASIC complexes to acid signaling in neurons.

## Background

Acid signaling in the brain has attracted increasing attention in recent years. In physiological conditions, protons are now recognized to function as neurotransmitters [1–4]. In neurological diseases such as ischemia, brain acidosis is an important contributor to neuronal injury [5–7]. The main neuronal proton receptor is the acid-sensing ion channel (ASIC) [8–10]. In the brain, both in situ hybridization and immunostaining data show that ASIC1a, 2a and 2b subunits are predominantly expressed in neurons [9, 11–14]. It is well established that ASIC1a is one important mediator of acid-activated responses in neurons and acidosis-induced changes in disease [15, 16]. Although acid-activated currents in multiple neurons show components from both ASIC1a homomers and 1a/2 heteromers [17–24], the relative contribution of different configurations of ASIC channels to acid signaling in the brain remains unclear. Answering this question is essential for a better understanding of how ASICs regulate neuron function in physiological and pathophysiological conditions.

One recent report examined the composition of ectopically expressed 1a/2a heteromers in *Xenopus* oocytes [25]. The result indicated that the proportion of ASIC1a homomers, 2a homomers, and 1a/2a heteromers (at either 2:1 or 1:2 stoichiometry) mainly depended upon the relative expression of the subunits, and that there was no preferential assembly into a specific type or stoichiometry [25]. In another study, Baron et al. transfected COS cells with ASIC1a + ASIC2a at different cDNA ratios [26]. Increasing 1a:2a cDNA ratio from 1:1 to 2:1 shifted pH_50_ from 5.5 to 6.05 and reduced the desensitization rate by 61 %. Although the relative protein ratio was unknown in the Baron study, the Baron and Bartoi data together suggested that the relative molar ratio of various ASIC subunits is the critical determinant of ASIC channel composition in the brain. Given the unique pharmacological properties of homomeric and heteromeric ASICs, obtaining quantitative information on relative ASIC subunit expression in the brain is critical for efficient targeting of ASIC channels in disease.

Here, we used a biochemical approach and semi-quantitatively determined the relative molar ratio of various ASIC subunits in the brain. Further, we asked whether differential expression of ASICs in different brain regions determines region-specific surface trafficking of ASIC1a, 2a, and 2b.

## Results

### A method to semi-quantitatively assess ASIC subunit ratio in the brain

**Fig. 1:**
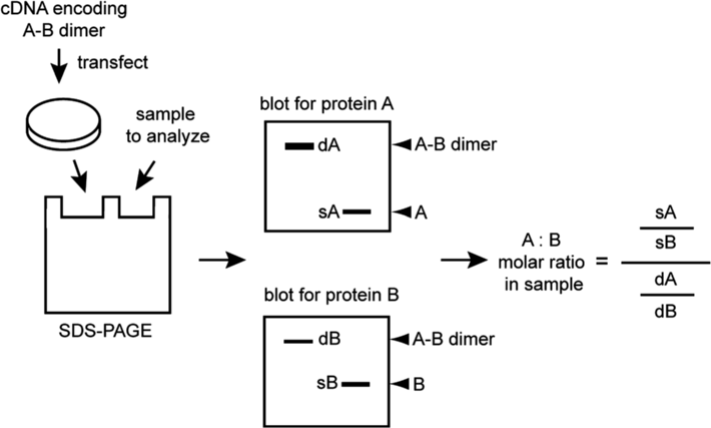
A method to determine relative molar ratios of two proteins. To facilitate quantitative measurement of relative molar ratio of two proteins, A and B, an A-B dimer construct is generated and expressed in cells. The lysate from these cells and samples of interest are loaded onto gels, and blotted for A and B separately. Since the A-B dimer contains A and B at a 1:1 molar ratio, the ratio obtained from the dimers on two blots, dA / dB, serves as a calibrator for the ratio obtained from the sample, sA / sB

To quantitatively compare the molar ratio of two proteins, we presented here a biochemical calibration method (Fig. 1). For any two protein A and B, we will generate a cDNA construct encoding an A-B dimer, and express the fusion construct in cells. We will run the lysates from A-B dimer expressing cells together with the biological samples of interest, and blot for proteins A and B. The dimer protein contains A and B at a 1:1 molar ratio. Thus, the signal intensity obtained from the dimers (dA and dB as drawn in the left lanes on the illustrated blots) serves as a calibrator for the signals obtained from the samples of study. The ratio for the two proteins from the test sample (sA and sB as illustrated in Fig. 1) is then calibrated with the ratio dA/dB. The calibrated ratio represents the absolute molar ratio of the two proteins in the test sample.

**Fig. 2:**
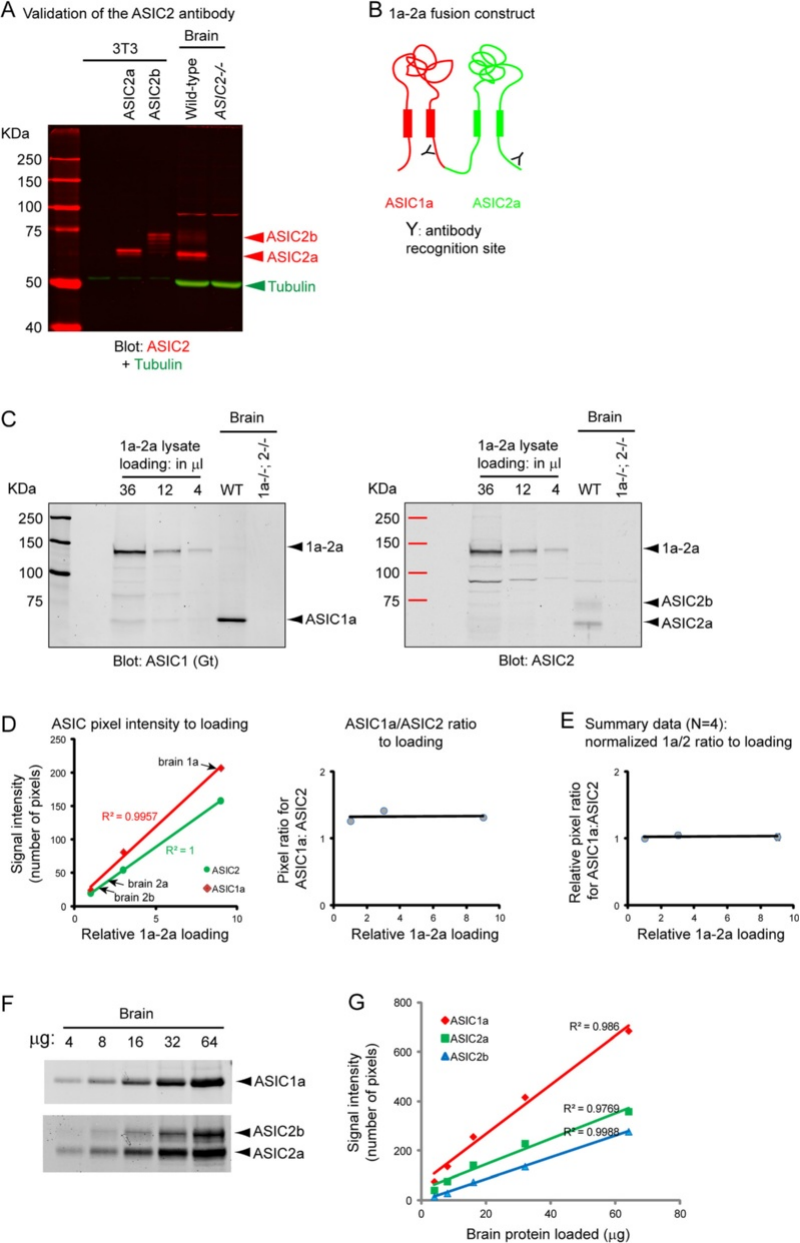
Validating the method for studying molar ratio of ASICs in the brain. **a** Specificity of an ASIC2 antibody. 3T3 cells transfected with ASIC2a or ASIC2b and brains from wild-type (WT) or ASIC2-/- mice were blotted with a rabbit ASIC2 IgG (*red*) and a mouse Tubulin (*green*) antibody, and detected simultaneously using a laser scanning imaging system (Li-Cor). Arrowheads on the right indicate the relative position of the indicated proteins. Note specific ASIC2a (~66KD) and ASIC2b (~75KD) bands were present in wild-type but not in ASIC2-/- brain lysates. **b** Diagram showing the ASIC1a-ASIC2a (1a-2a) fusion construct and the relative position of the region recognized by the antibodies used. **c** Blots showing the expression of the 1a-2a dimer and brain ASICs. Left three lanes were loaded with various amounts of lysate from cells overexpressing the 1a-2a fusion construct (runs at ~130KD). The blot was blotted for ASIC1a, ASIC2, and tubulin at the same time. **d** Quantification showing the density (*left graph*) of ASIC1a or 2a in (**c**) and the ratio (right graph) of ASIC1a:ASIC2 pixels with different levels of loading. **e** Summary data for the change of 1a:2 ratio obtained for the 1a-2a dimer at different loading. In order to compare between experiments, the ratio at the lowest loading was arbitrarily set to 1 in each experiment. Note that when we calibrated 1a/2 ratio for the brain proteins, we used the raw ratio obtained in panel G as the calibrator (see Results for further elaboration). (**f** & **g**) Blots (representative from 4 repeats) and quantification showing the signals of ASIC1a, 2a, and 2b at loadings between 4 and 64 μg of total brain protein

In order to apply this approach to study ASIC expression in vivo, we raised an ASIC2 antibody against the last 20 amino acids of ASIC2. Figure 2a illustrates the specificity of this antibody. ASIC2a (512 amino acid) and 2b (563 amino acid) migrated with an apparent molecular weight of about 66 and 75 KD, respectively. Since ASIC2a and 2b are identical at their C-terminal region [13], the relative intensity of ASIC2a and 2b on the gel reflects the relative molar ratio of these two subunits. In addition to this rabbit anti-ASIC2, we used a goat anti-ASIC1a (see Fig. 2c for specificity of this antibody).

Next, we engineered an ASIC1a-ASIC2a fusion construct (Fig. 2b) and transfected it into CHO-K1 or 3T3 cells. We loaded different amounts (at a ratio of 9: 3: 1) of lysate obtained from 1a-2a expressing cells, blotted for goat anti-ASIC1a and rabbit anti-ASIC2, and quantified the signal intensity from cells overexpressing the 1a-2a fusion construct (Fig. 2c). The signals (in raw pixels) of both ASIC1a and 2a fit into a perfect linear relationship with the loaded amount (Fig. 2d, left panel).

To assess how loading affects the detected 1a/2 ratio obtained from the 1a-2a dimer, we plotted the 1a/2 ratio for the 1a-2a dimer over loading. The right panel in Fig. 2d showed the result from a single experiment. The 1a/2a ratio of the 1a-2a dimer was largely unchanged over the 9 fold range. We then quantified the 1a/2a ratio of 1a-2a dimers at different loadings from multiple experiments (*N* = 4). Due to the differences in blotting, the absolute 1a/2a ratio for the dimers varied from experiment to experiment. Therefore, to compare multiple experiments, we normalized the ratio in each experiment to that of loading 1 in that experiment. Fig. 2e showed the summary data: the 1a/2a ratio remained constant over the 9 fold loading range. This result indicates that a single point calibration is sufficient for a semi-quantitative determination of ASIC1:2 subunit ratios in the brain.

Similar to what is shown in Fig. 1, to calculate absolute ASIC1a:ASIC2 ratio in brain samples, we used the following equation:

$$ \left(\frac{brain\kern0.5em  ASIC1a}{brain\kern0.5em  ASIC2a\kern0.5em  or\kern0.5em 2b}\right)/\left(\frac{ASIC1a\kern0.5em  from\kern0.5em 1a-2a\kern0.5em  dimer}{ASIC2\kern0.5em  from\kern0.5em 1a-2a\kern0.5em  dimer}\right) $$

One important note is that, when we calculate the expression ratio from the brain, we did not use the “normalized” ratio in Fig. 2e. Instead, we used the raw pixel ratio for the 1a/2a dimer obtained from the dimers on the same blot. For example, when we quantified ASIC protein of the WT lanes in Fig. 2c, we used the ratio obtained from the right panel of Fig. 2d.

At 20 μg loading, the pixels for brain ASIC1a were at the top, while those for brain ASIC2 were at the bottom, of the range obtained from the 1a-2a dimer (see arrows in Fig. 2d, left panel). Regarding whether these signals from brain ASICs were at the limit of the linear range of the scanner, we loaded brain proteins at 4, 8, 16, 32, and 64 μg per lane, blotted for ASIC1 and ASIC2, and analyzed the relative pixel intensity of ASIC1a, 2a, and 2b over the loading amount (Fig. 2f, g). The signals for all three ASICs fit into a good linear relationship between 4 and 64 μg. These data indicate that ASIC signals from 20 μg of brain lysate remained within a good linear range of the detector. In addition, the slopes of ASIC2a and 2b were similar to each other. This result verified that ASIC2a and 2b, although running at different positions, exhibited a similar linear relationship with loading when detected with our ASIC2 antibody.

### ASIC1a subunits outnumber ASIC2a + 2b subunits in the brain

**Fig. 3:**
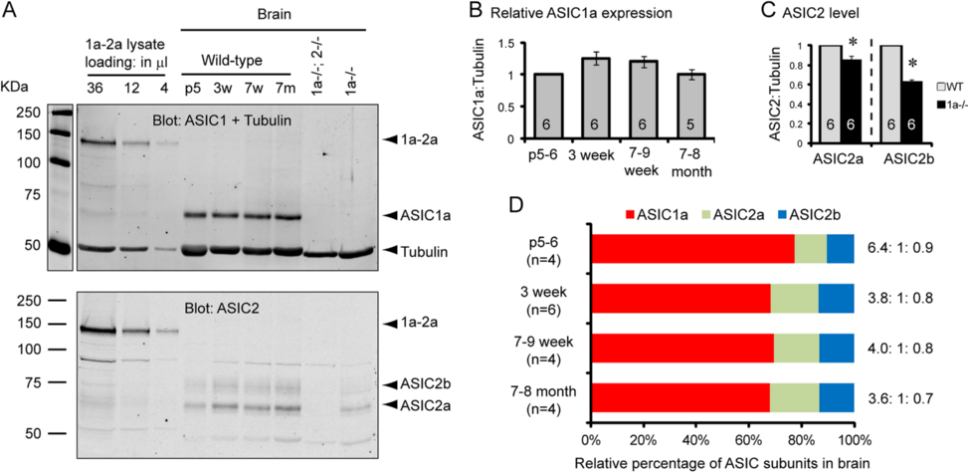
Developmental changes in ASIC expression in the brain. **a** Representative Western blots showing ASIC1a, 2a, 2b expression at different postnatal stages. Blots shown are representative from 4 separate repeats. **b** Quantification showing the relative ASIC1a expression level at different age. Numbers on the bars are number of animals analyzed. **c** Quantification of relative ASIC2a and 2b protein level in WT and ASIC1a-/- brain. Asterisks indicate significant differences (*p* = 0.009 for ASIC2a, *p* < 0.001 for ASIC2b, Student's *t*-test). **d** Quantification of relative expression of ASIC1a:2a:2b in the brain at different postnatal ages. Since we normalized the ratio using the 1a-2a construct, the numbers obtained represent the ratio of the number of subunits

With the above method, we examined developmental changes in ASIC expression in the brain. Compared to P5-6, the ratio of ASIC1a: tubulin increased by 25 and 21% at 3 week and 2 month old, respectively (Fig. 3a, b), although the difference was marginally (*p* = 0.0496 for 3 week vs. P5–6; *p* = 0.072 for 7–9 week vs. P5–6). The blots also suggested that ASIC1a-/- brain had less ASIC2. We quantified the levels of ASIC2a and 2b in WT and ASIC1a-/- brain, and found that both ASIC2a and ASIC2b levels were reduced in the ASIC1a null brain (Fig. 3c). We next quantified the relative ASIC1a, 2a and 2b ratio in the brain. At all ages examined, there were more ASIC1a than ASIC2a + 2b (Fig. 3a, d). After calibrating using the signals from 1a-2a, we found that the relative ASIC1a:2a:2b ratio was 6.1:1:0.86 at postnatal day 5–6. The relative amount of ASIC2 increased at weanling, and the ratio of 1a:2a:2b declined to about 3.8:1:0.75. This ratio remained constant at the rest of time points tested (up to 8 month of age). These data demonstrate that ASIC1a constitutes two-third or more of total ASIC subunits at all ages examined.

**Fig. 4:**
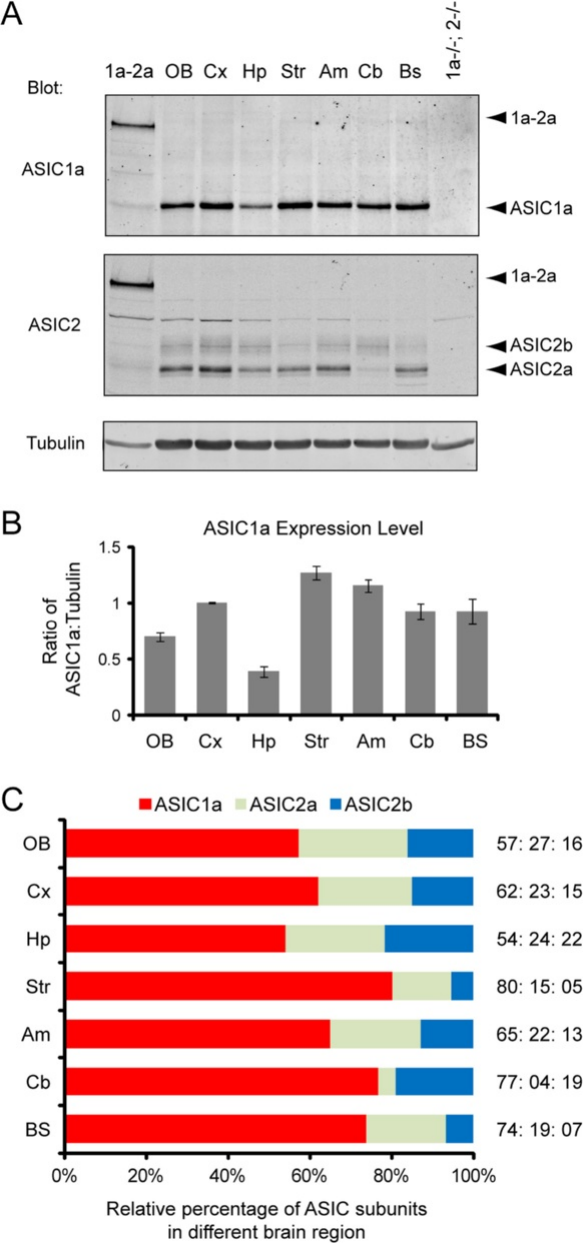
Differential expression of different asic subunits among brain regions. **a** Representative Western blots showing the expression of ASIC1a, ASIC2a and ASIC2b in different brain regions of 7–9 week old mice. **b** Quantification showing the relative ASIC1a expression level (ASIC1a:tubulin ratio) in different brain regions. **c** Quantification showing the relative ASIC1a:2a:2b ratio in different brain region. *N* = 5 animals. Abbreviations: Ob olfactory bulb; Cx cerebral cortex; Hp hippocampus; Str striatum; Am amygdala; Cb cerebellum; Bs brain stem

Next, we studied the expression of ASICs in different regions of the adult brain (Fig. 4a). Striatum, amygdala and cortex showed relatively higher levels of ASIC1a, while hippocampus and olfactory bulb had lower levels of expression (Fig. 4b). The percentage of ASIC1a subunits ranged from 54 % in hippocampus to 80 % in striatum. Hippocampus also exhibited the most balanced expression of all three subunits; the ASIC1a:2a:2b ratio in hippocampus was 54:24:22 (we presented the ratio in this manner so that the total number of three subunits equals 100). Cortex, olfactory bulb and amygdala also had similar levels of ASIC2a and 2b expression. In contrast, cerebellum showed little ASIC2a expression but had significant levels of ASIC2b; 1a:2a:2b ratio in cerebellum was 77:4:19. The majority of ASIC2 in striatum and brain stem, on the other hand, were ASIC2a. A detailed summary of the result is presented in Fig. 4c.

### Differential trafficking of ASIC subunits to the cell surface

**Fig. 5:**
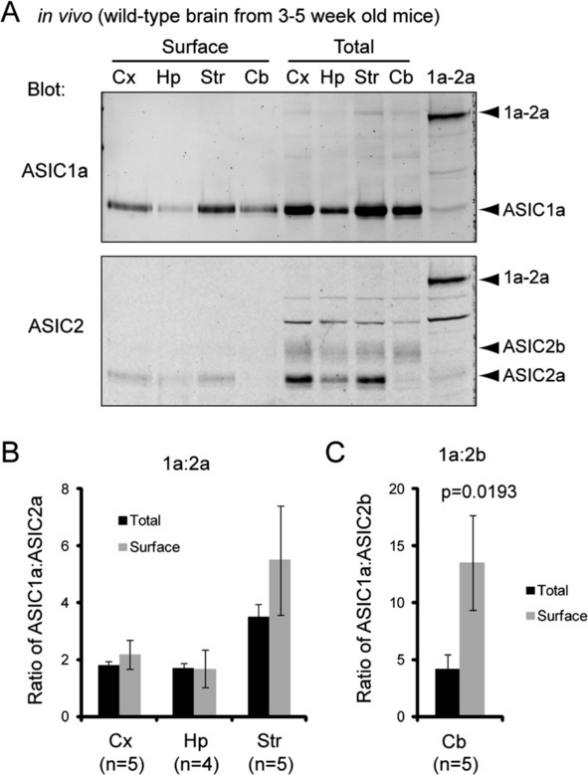
Differential surface trafficking of ASIC1a, 2a, and 2b in the brain. **a** Western blot showing surface and total ASIC levels in acutely dissected brain tissue. Different brain regions (as indicated) were dissected from 3 to 5 week old mice. Surface fractions were isolated by surface biotinylation and NeutrAvidin pull down as described in Methods. Membranes were blotted for ASIC1a and ASIC2. **b** & **c** Quantification showing ASIC1a:2a (**b**) and 1a:2b (**c**) ratio in the corresponding brain regions. We quantified 1a:2b ratio for cerebellum because there was little ASIC2a in cerebellum

Typically only a fraction of proteins reach cell surface, and this surface fraction determines the functional contribution of an ion channel. Therefore, we asked whether surface ASICs in the brain exhibit a similar subunit ratio as their total expression. We performed surface biotinylation on acutely dissected brain tissues. We focused our analysis on four brain regions with distinct patterns of ASIC2 expression (see Fig. 4c): cerebellum (expresses mostly 2b), striatum (expresses mostly 2a), hippocampus and cortex (express similar levels of 2a and 2b). While all regions express high levels of ASIC1a, comparing these four regions will be informative regarding understanding the specific role of ASIC2a vs. 2b on surface trafficking in the brain. Surface levels of ASIC1a and 2a changed in parallel to their total expression (Fig. 5a, b). There were no significant differences in ASIC1a:2a ratio between surface and total fractions, with ~2:1 ratio for hippocampus and cortex and ~4:1 ratio for striatum. In contrast, in all four regions, we detected little ASIC2b at the surface (Fig. 5a). For cerebellum, we quantified the 1a:2b ratio because it expresses little ASIC2a. Consistent with a diminished surface 2b level, 1a:2b ratio at cell surface in cerebellum was significantly higher than that in total lysate (Fig. 5c).

**Fig. 6:**
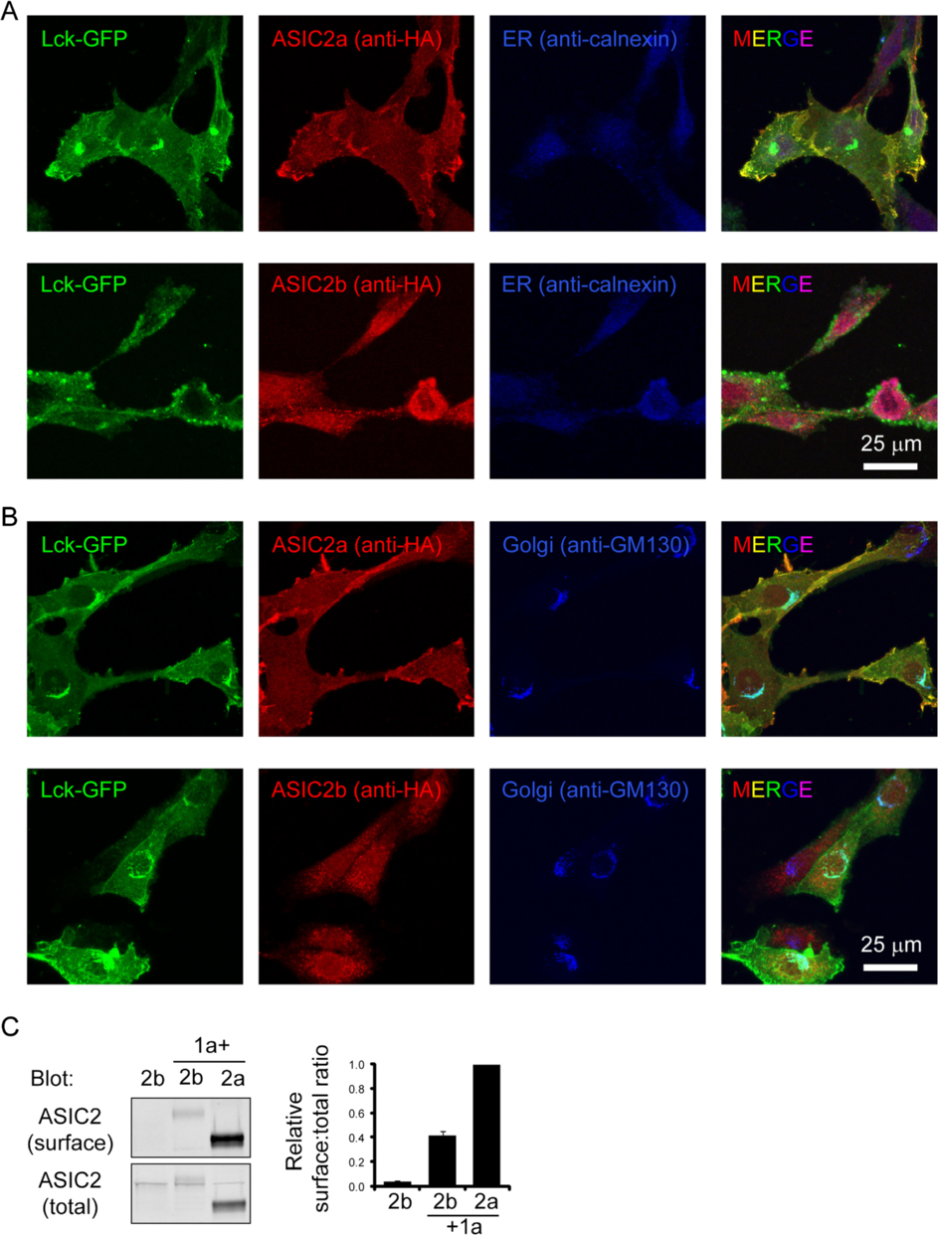
ASIC2a shows membranous localization while ASIC2b localizes to ER in 3T3 cells. **a** & **b** Representative confocal images showing ASIC2a and 2b localization in 3T3 cells. 3T3 cells were transfected with HA-tagged ASIC2a or 2b, together with a membrane-targeted Lck-GFP. Localization of ASIC2 was detected with anti-HA; ER and Golgi was detected with anti-calnexin (**a**) and anti-GM130 (**b**), respectively. ER and Golgi was detected in the far red channel but pseudocolored in blue. **c** Western blot and quantification of ASIC2b and 2a surface expression in 3T3 cells. 3T3 cells were transfected with ASIC2b alone, ASIC2b together with 1a, or ASIC2a together with 1a. Surface proteins were biotinylated and analyzed as described in Methods. The three conditions show significant differences from each other (*p* < 0.001, *N* = 4)

This result suggests that ASIC2b does not traffic as efficiently as ASIC2a to cell surface. To determine whether ASIC2b preferentially localizes to one specific subcellular compartment, we studied localization of ASIC2 in heterologous cells. ASIC2a exhibited a lower level of diffuse staining inside the cell and a higher level of staining on the edge of the cell, overlapping to a large part with the signal of Lck-GFP, a membrane-targeted GFP (Fig. 6a, upper panel). This pattern is typical for a membrane protein, and consistent with one earlier report [27]. In contrast, ASIC2b was predominantly intracellular and co-localized with an endoplasmic reticulum marker (Fig. 6a, lower panel). We also stained for Golgi with anti-GM130. Neither ASIC2a nor ASIC2b showed significant overlap with the Golgi marker (Fig. 6b).

Next, we asked whether co-expressing ASIC1a with 2b increases ASIC2b surface expression. We transfected 3T3 cells with ASIC2b, alone or together with ASIC1a, performed surface biotinylation, and blotted surface and total proteins for ASIC2. Consistent with data from the brain and immunostaining in cells, when expressed alone, ASIC2b was barely detectable at cell surface (Fig. 6c). Co-expressing ASIC1a significantly increased surface 2b level. However, when co-expressed with ASIC1a, 2b surface:total ratio remained lower (42 % ± 3 %, *p* = 0.003) as compared to 2a.

## Discussion

We determined here ASIC subunit ratio with a biochemical approach that can determine the absolute molar ratio of any two given proteins. Of note, a similar approach has been used previously to probe channel stoichiometry of other ion channels [28]. The only requirement of this approach is the availability of specific antibodies to such endogenous proteins. Compared to other semi-quantitative methods such as Mass Spectrometry, this method is simple and does not require special instruments. More importantly, this method is feasible to study low abundance proteins, including most ion channels and receptors. Ratiometrically, we performed semi-quantitative measurement of ASIC subunit ratios in brain. As elaborated below, although there are clearly sub-region and cell-type specific variations, the data indicate that the main functional ASICs in the brain are ASIC1a homomers and 1a/2a heteromers at a 2-1a:1-2a stoichiometry. Regionally, our data show specific differences in ASIC2a and 2b expression, and suggest several functional differences between ASIC2a and 2b *in vivo*. These results have important implications in interpreting how ASICs mediate acid signaling *in vivo* as well as for future targeting of ASICs in disease.

ASICs function as homomeric and heteromeric trimers [29–31]. In all brain regions examined, ASIC1a subunits constituted more than half of the three (1a, 2a, and 2b) ASIC subunits examined. It is clear that there are variations within each region and between different types of neurons. However, to provide some insight into the overall proportion of ASIC1a homomers and 1a/2a heteromers, we performed a modeling based on two simplified assumption. First, based on a previous study which shows that the assembly of ASIC1a and 2a homomers or heteromers has no apparent preference of a specific complex or stoichiometry [25], we assumed that the assembly of ASIC channels was mostly dependent upon the relative number of subunits. Second, we assumed a random distribution of different ASIC subunits within each region. Although these assumptions are clearly over-simplified, they allow us to estimate the relative proportion of various ASIC channels at a global level. Our calculations (see Table 1) suggest that: the main functional ASICs in the brain are ASIC1a homomers and 1a/2a heteromers, the number of ASIC2a homomers is negligible, and the overall 1a/2a heteromers favor a 2-1a: 1-2a stoichiometry. In addition, other than striatum and cerebellum, where ASIC1a homomers are dominant, 1a/2a heteromers constitute ≥ 50 % of all functional ASICs. Although a specific neuron may deviate significantly from the estimated proportion, our data indicate that 1a homomers and 2-1a:1-2a heteromers are favorable complexes for future drug targeting of ASICs in the brain.

**Table 1:** A simplified prediction (assuming homogenous distribution of ASICs within a region) of the relative proportion of ASIC1a homomers, 1a:2a heteromers and 2a homomers in different brain regions

Relative % of ASIC subunits	OB	Cx	Hp	STR	Am	Cb	BS
ASIC1a	57.4 %	62.2 %	54.3 %	80.1 %	65.0 %	76.8 %	73.9 %
ASIC1b	26.6 %	23.0 %	24.1 %	14.6 %	22.2 %	4.3 %	19.3 %
ASIC1c	16.0 %	14.9 %	21.7 %	5.3 %	12.8 %	19.0 %	6.7 %
Relative & of functional ASICs							
1a:1a:1a	31.9 %	38.9 %	33.2 %	60.5 %	41.5 %	85.0 %	49.8 %
1a:1a:2a	44.4 %	43.2 %	44.2 %	33.1 %	42.4 %	14.2 %	39.1%
1a:2a:2a	20.5 %	16.0 %	19.6 %	6.0 %	14.5 %	0.8 %	10.2 %
2a:2a:2a	3.2 %	2.0 %	2.9 %	0.4 %	1.6 %	0.0 %	0.9 %

Top three rows are the percentage of 1a, 2a and 2b subunits, based on Fig. 4. The lower four rows are the proportion of 1a homomers and 1a:2a heteromers. The proportions are calculated based on total protein expression and assuming a random assembly of ASIC subunits into trimers [25]. Since we detected little ASIC2b at cell surface in the brain, we did not include ASIC2b-containg complexes in this table for simplicity. Initial percentage was calculated as follows

1a homomer = %1a × %1a × %1a

2a homomer = %2a × %2a × %2a

1a: 2a heteromer (2:1 ratio) = %1a × %1a × %2a × 3

1a:2a heteromer (1:2 ratio) = %1a × %2a × %2a × 3

To calculate the relative percentage of each of the four trimers, the raw percentage was then divided by the total percent of the four trimers.

We focus our study here on ASIC1a and ASIC2 but inquire regarding the likelihood of other ASIC contributions to acid-activated currents in brain. ASIC1b is primarily expressed in DRG [32]. ASIC3 is also mostly expressed in the periphery, and its expression in brain is limited to the sensory mesencephalic trigeminal nucleus [33–36]. ASIC4, which by itself does not conduct current, is detected in multiple brain regions but its expression is limited to a subset of interneurons, NG2 positive glia, and cerebellar granule cells [37–39]. Overall, most brain neurons express ASIC1a, 2a and 2b [11–13]. The wide expression of ASIC1a and ASIC2 in various types of brain neurons also fits with electrophysiological recordings (see below). Thus, our data have general implications for acid-activated responses in the vast majority of neurons in brain.

As shown in Table 1, our data predict that ASIC1a homomers constitute to ~85 % of functional ASICs in cerebellum. In both cerebellar granule neurons and Purkinje cells, the acid-activated currents show a predominant contribution from ASIC1a homomeric channels [17, 40]. In striatum, the main class of neurons is medium spiny neuron. These neurons show current properties suggestive of 70.5 % ASIC1a homomers and 29.5 % 1a/2a heteromers [20]. These numbers correlate well with our prediction, which shows that, in striatum, ASIC1a homomers, 2-1a:1-2a, and 1-1a:2-2a heteromers, constitute to 60.5 %, 33 %, and 6 % of functional ASICs, respectively. In addition, acid-activated currents in neurons from other brain regions, including hippocampus, cortex and olfactory bulb exhibit a mixed contribution from ASIC1a homomers and 1a/2a heteromers [18, 21, 23]. Again, these results fit with our prediction (Table 1). Accordingly, our ratiometric and regional data are consistent with previous electrophysiological recordings and provide a molecular and semi-quantitative explanation for the observed current properties. In addition, the developmental increase in ASIC2:1 ratio in brain (Fig. 3) constitutes the same trend as that observed during the maturation of cultured neurons [41]. The difference in 1a/2a/2b ratio in the brain suggests changes in current properties. One property of particular interest is steady state desensitization. ASIC1a homomers and 1a/2b heteromers exhibit steady state desensitization with mild acidosis [22, 42–45]. Our data here suggest that chronic acidosis has differential effects on neurons in different brain regions. In addition, both inhibitory and excitatory neurons exhibit ASIC currents [46, 47]. Therefore, the exact outcome of ASIC activation in the brain depends on the type of neurons that get activated.

The low level of ASIC2b at the cell surface is a somewhat unexpected finding as a previous study reported that, in about 50 % of dissociated hippocampal neurons, acid-activated current contains a significant contribution which resembles that of ASIC1a/2b heteromers [22]. We speculate that this discrepancy could be due to the difference in the experimental system: acute brain tissue in our study vs. dissociated neuron in the previous one. This is partially supported by the observation that there were detectable surface ASIC2b in 3T3 cells, especially when co-expressed with ASIC1a. Nevertheless, due to a diminished surface presence in brain tissue, ASIC2b probably has little direct contribution to the amplitude of ASIC currents in brain. These data indicate that the primary function of ASIC2b is to modulate ASIC1a expression and serve as an intracellular retainer for subunits associated with it. It will also be interesting to investigate whether ASIC2b expression alters ASIC1a/2a ratio in the brain, and whether pathological conditions alter ASIC2b trafficking to cell surface.

## Conclusions

In summary, with a simple biochemical approach, we performed a semi-quantitative quantification of relative ASIC1a and 2 subunits in the brain. Our results indicate that ASIC1a constitutes to more than 50 % of all three ASIC subunits throughout the brain. ASIC1a and 2a exhibit efficient surface trafficking while little ASIC2b reaches cell surface in brain tissues. Together, these results argue that the major functional ASICs in the brain are ASIC1a homomers and ASIC1a/2a heteromers (mostly at a 2-1a:1-2a stoichiometry). These data provide fundamental information for interpreting how different ASICs contribute to proton signaling in physiology and disease.

## Methods

### Mice

ASIC1a-/- and ASIC2-/- mice on a congenic C57/BL6 background were kindly provided by Drs. Michael Welsh and John Wemmie. *ASIC1a-/-*;ASIC2-/- double null mice were generated by cross-breeding the single knockouts. Genotype was verified by PCR (also see Western blots in Fig. 1). Wild-type and knockout mice were maintained as described earlier [48]. Postnatal day 5–9 (P5-9) pups or 3 week to 8 month old adults (either sex unless specified) were used. Animal care met National Institutes of Health standards and all procedures were approved by the Animal Care and Use Committee at University of South Alabama.

### Constructs and reagents

Constructs encoding mouse ASIC2a, ASIC2b and N-terminal HA-tagged versions have been described earlier [31, 48–50]. The ASIC1a-ASIC2a (1a-2a) fusion construct was generated by PCR mediated subcloning, adding the full length mouse ASIC2a immediately following the last amino acid of mouse ASIC1a. All constructs used the same vector backbone of eGFP-c1, replacing eGFP with ASIC. All constructs contained the same Kozak sequence GCCACCATG, and were verified by sequencing. Other reagents used: NHS-sulfo-LC-biotin and NeutrAvidin beads (Pierce); *N*-ethylmaleimide (NEM, from Sigma); proteinase inhibitor cocktail (Roche); culture media and serum (HyClone and Invitrogen).

### Antibodies

We raised raised five rabbit ASIC2 antibodies using the following synthetic peptides: PGDAPYCTPE, CFDYIYELIKEKLLD, CETISHTVNVPLQTA, SHTVNVPLQTALGTLEEIAC (SHT), and VPLQTALGTLEEIAC (VPL). These peptides were conjugated to KLH (antisera generated by Syd Labs). The antibodies raised with the SHT (see Fig. 2a) and VPL (not shown) peptides were specific in detecting corresponding endogenous ASICs. The SHT peptide yielded a superior ASIC2 antibody, and was used in all experiments presented here. The IgG fraction, affinity purified by passing through a Protein G-agarose column, was used for blotting. When blotting for ASIC1a and 2 together, we used the rabbit ASIC2 (SHT) antibody together with a goat anti-ASIC1 (Santa Cruz, SC-13905, see Fig. 2c for specificity). Other primary antibodies used: mouse anti-tubulin (University of Iowa Developmental Hybridoma Bank and Sigma). Secondary antibodies used: Alexa 649-, 680-, 800- and Dylight 680-, and 800-conjugated secondary antibodies (Invitrogen, Li-cor, and Pierce).

### Cell culture and transfection

CHO-K1 and NIH 3T3 cells were purchased from ATCC. CHO-K1 cells were cultured in F12K, supplemented with 10% fetal bovine serum. 3T3 cells were cultured in DMEM hi-Glucose, supplemented with 10 % fetal calf serum. Lipofectamine 2000 mediated transfection was performed in a manner similar to that described earlier [51].

### Surface biotinylation and neutravidin pull-down

Surface biotinylation in cells was performed as described earlier [51, 52]. For surface biotinylation of endogenous proteins in the brain, various brain regions were isolated in cold HBSS + 6mg/ml glucose. In the initial experiments, we sliced the tissue to 200 μm thickness with a tissue chopper. In later repeats, we manually chopped the tissue into small pieces, and generated similar surface labeling result as slicing. The data obtained from slicing and manual chopping were pooled and analyzed together. All the following procedures were carried at 4 °C or on ice: the slices/tissue were transferred to a 2 ml tube containing 1 ml of ice-cold PBS+/+, centrifuged for 15–30 s at 500 rpm and the supernatant discarded. 1.5 ml of Sulfo-NHS-LC-Biotin (0.5–1 mg/ml in PBS) was added to the tissue and the tubes were rotated for 40–45 mins at 4 °C. The tissue was spun down, and rotated at 4 °C for 5–10 min in PBS with 100 mM glycine (to quench unreacted biotin). Biotinylated slices were lysed in lysis buffer (PBS 1 % triton X-100 or PBS 1 % triton X-100 0.5 % SDS) with freshly added proteinase inhibitors (1.2x) and 1 mg/ml NEM. NeutrAvidin pulldown was performed as described earlier [51, 53].

### Brain lysate and western blotting

Whole brain or biotinylated brain slices were homogenized in lysis buffer with freshly added protease inhibitors and 1 mg/ml *N*-ethylmaleimide (NEM). Lysates were sonicated briefly and cleared by centrifugation. Protein concentration was quantified using a Bio-Rad RC-DC kit. Equal amounts (typically 20 μg) of brain lysate were loaded per lane for Western blot.

The samples were separated by 8 % or 10 % SDS-PAGE and transferred to nitrocellulose membranes. Blotting was performed according to instructions of the Odyssey Imaging System (Li-cor). Antibody dilutions were: goat anti-ASIC1 1:1000; rabbit anti-ASIC2 IgG 1:500; monoclonal anti-tubulin 1:30–60K; secondary antibodies were used at 1:10–16K. Blots were imaged using an Odyssey Infrared Imaging System according to manufacturer’s instructions. Densitometry of imaged bands was performed in NIH ImageJ as described earlier [49, 50].

### Immunofluorescence and confocal microscopy

For immunofluorescence, CHO cells were initially transfected with HA-tagged ASIC2a or ASIC2b together with a membrane-targeted Lck-GFP in 35 mm dishes and re-plated into 4 well chamber glass slides 1 day after transfection. Similar to what has been describe earlier [54], ASIC2 was detected with a rat anti-HA antibody (Roche, #11867423001, 1:1000 dilution). ER and Golgi was detected with a mouse anti-calnexin (BD biosciences, #610523, 1:500 dilution) and a mouse anti-GM130 (BD biosciences, #610822, 1:500 dilution), respectively. Secondary antibodies used were Dylight 568-conjugated donkey anti-rat and Dylight 649-conjugated donkey anti-mouse antibodies. Confocal images were captured using a Nikon A1 laser scanning microscope. Illumination was provided by an argon (Ar, 458, 488, 514 nm lines) and two diode (561 and 640 nm lines) lasers. Green, red and far red channels were imaged sequentially. Images were captured with a 63×/1.2NA PL APO water lens. Images were exported and further processed with Adobe Photoshop as described previously [51].

### Statistical analysis

For comparisons between two groups, we used student’s *t*-test. For multiple comparisons, we used ANOVA with a Bonferroni or Turkey’s HSD test. Data were reported as mean ± s.e.m. for the number of samples indicated.

## Abbreviations

ASIC: acid-sensing ion channel
CHO: Chinese hamster ovary
ER: endoplasmic reticulum
HA: hemagglutinin
KO: knockout
WT: wild-type
